# Association Between Acute Medical Exacerbations and Consuming or Producing Web-Based Health Information: Analysis From Pew Survey Data

**DOI:** 10.2196/jmir.3801

**Published:** 2015-06-23

**Authors:** Risha Gidwani, Donna Zulman

**Affiliations:** ^1^ VA Health Economics Resource Center Menlo Park, CA United States; ^2^ VA Center for Innovation to Implementation Menlo Park, CA United States; ^3^ Division of General Internal Medicine Stanford University Stanford, CA United States

**Keywords:** Internet, health knowledge, attitudes, practice, emergencies, hospitalization

## Abstract

**Background:**

The Internet is an increasingly important resource for individuals who seek information from both health professionals and peers. While the demographic and health characteristics of persons who use health information technology has been well described, less is known about the relationship between these health characteristics and level of engagement with health information technology. Even less is known about whether persons who produce Web-based health information differ in health status from persons who consume such content.

**Objective:**

We explored the health characteristics of persons who engage with the Internet for the purposes of consuming or producing Web-based health information, and specifically, whether healthier versus sicker persons engage with health information technology in different ways.

**Methods:**

We analyzed data from the 2012 Pew Health survey, a landline and cell phone survey of 3104 adults in the United States. Using multiple logistic regression with sampling weights, we examined the association between sociodemographic and health characteristics and the consumption or production of Web-based health information. Sociodemographic variables included age, sex, race, and education. Health characteristics included self-reported health status, presence of chronic condition(s), and having an acute medical exacerbation. Acute medical exacerbations were defined as an emergency department visit, hospitalization, or other serious medical emergency in the last 12 months.

**Results:**

The majority of the sample reported good or excellent health (79.7%), although 50.3% reported having at least one chronic condition. About a fifth (20.2%) of the sample experienced an acute medical exacerbation in the past year. Education was the sociodemographic characteristic most strongly associated with consuming Web-based health information. The strongest health-related predictors of consuming Web-based health information were an acute medical exacerbation (OR 2.39, *P*<.001) and having a chronic condition (OR 1.54, *P*=.007). Having an acute medical exacerbation was the only predictor of producing Web-based health information (OR 1.97, *P*=.003). All participants, regardless of health status, were most interested in Web-based health information regarding diseases or medical problems. However, persons with acute medical exacerbations were more likely to seek Web-based health information regarding medical tests, procedures, and drugs compared to persons without acute medical exacerbations.

**Conclusions:**

Producers of Web-based health information differ from consumers of this information in important health characteristics that could skew the content of peer-generated Web-based health information and overrepresent the experiences of persons with acute medical exacerbations. Providers may have a role to play in directing patients towards high-quality, easy-to-understand online information, especially information regarding treatments and procedures.

## Introduction

The Internet has become a key source of health information for many individuals. In 2012, 59% of adults looked for health information online [1]. A wide variety of sources are being searched for Web-based health information, including health care systems [2-4], government agencies [5,6], non-profit/for-profit organizations [7,8], and disease-specific advocacy groups [9-11].

Increasingly, patients are turning to other patients as trusted sources of information [12,13]. Some patients have developed websites or blogs where they share their experiences living with chronic conditions [14,15]. Others review medical treatments, procedures, health care products, and hospitals and providers online [16,17]. Individuals often seek patient-generated information after medical appointments, especially if their clinic visits generated anxiety or dissatisfaction [18]. However, while patient-generated health information can provide a unique and valuable perspective for others with shared medical issues or health care challenges, there are also concerns about the potential for dissemination of inaccurate or incomplete information [16,19]. Typically, a small number of super-users post the vast majority of content [12,13,20]. Little is known about the extent to which these individuals’ sociodemographic characteristics, health conditions, and health care experiences reflect those of the majority of patients consuming information.

In order to engage patients through the Internet, it is important that Web-based health information addresses patients’ specific needs and concerns and that sources are reliable, trustworthy, and relevant to the individuals seeking information. We sought to understand the circumstances under which patients consume and produce Web-based health information, and the influence of health status on these activities. Our objective was to understand the characteristics of Web-based health information consumers and especially the type of health-related information they sought online and to determine whether producers of health information represent the larger population of consumers with respect to sociodemographic and health characteristics.

## Methods

We used data from the Pew 2012 Health survey, sponsored by the Pew Research Center’s Internet & American Life Project, to evaluate predictors of engagement with Web-based health information. The English- and Spanish-language telephone (landline and mobile phone) survey was conducted with 3014 adults living in the United States. The survey collected self-reported data on persons’ Internet use, health status, and demographic characteristics.

Our primary outcome variable was use of the Internet to consume information about diseases or health conditions, which we refer to as “health-related Internet use”. A person was considered to have health-related Internet use if they reported using the Internet and reported looking online for any of the following in the previous 12 months: (1) a specific disease or medical problem, (2) trying to figure out what medical condition they or someone else might have, (3) a medical treatment or procedure, (4) how to lose or control weight, (5) a drug seen advertised, (6) drug safety or recall information, (7) any other health issue, (8) signing up for email updates regarding health or medical issues, (9) reading or watching someone else’s health- or medical issue-related commentary or personal experience, (10) finding others with similar health concerns, or (11) consulting online rankings or reviews of providers, hospitals, medical facilities, drugs, or medical treatments.

We also investigated the type of engagement people had with the Internet and specifically whether they were consumers or producers of Web-based health information. A participant was considered a “consumer” if they had health-related Internet use, as described above. An Internet user was considered a “producer” if they reported (1) posting information about their own personal health experience online, or (2) posting a review of a provider, hospital, experience with a particular drug, or experience with a medical treatment online. We examined participant characteristics associated with consumer versus producer behavior and the specific types of information that were consumed and produced. In post-hoc analyses, we also evaluated the specific types of Web-based health information consumed by persons with and without acute medical exacerbations, including the source of this information (peer, professional, or both).

All analyses employed multivariate logistic regression. In each analysis, we included age, highest level of education achieved (less than high school, high school, some college, or 4-year college or above), sex, race (white, Black, Asian/Pacific Islander, mixed, or Native American), self-reported health status, presence of chronic conditions, and an acute health exacerbation within the past year as covariates. Self-reported health status was classified as poor/fair, good, or excellent. Presence of chronic conditions was indicated if the participant reported having diabetes, high blood pressure, a lung condition, a heart condition, cancer, or “another chronic condition”. A participant was considered to have an acute medical exacerbation if they reported having an Emergency Department visit, a hospital visit, or a serious medical emergency in the previous 12 months. Income was not included in the model due to the high percentage of missing income data (18.7%) and concerns that income data were not missing at random.

All statistical analyses were conducted in Stata, version 12.0. Tests of significance were two-tailed and used an alpha of .05. Regression analyses employed survey sampling weights provided by Pew to account for a sample that was disproportionately stratified by race, differences in landline versus mobile phone response rates, and demographically based differential non-response.

## Results

### Overview

The majority of the sample was white (69.60%, 2098/3014) and insured (86.86%, 2618/3014) (Table 1). A little more than half of the sample was female (55.64%, 1677/3014); the mean age (SD) was 52.6 (19.8). Over one-third of the sample had a college degree or higher (36.99%, 1115/3014), and 8.93% (269/3014) had less than a high school education. Over a third (38.69%, 1166/3014) of the sample earned less than US $40,000 annually. However, high earners were also well represented in this sample; 13.70% (413/3014) made more than US $100,000 per year. The majority of the sample reported good or excellent health (79.66%, 2401/3014), although half (1498/3014) also reported having at least one chronic condition. About a fifth (20.21%, 609/3014) of the sample experienced an acute medical exacerbation in the past year. Of the 2392 persons who answered questions about health-related Internet use, 1717 (71.78%) reported consuming Web-based health information. Only 254 (10.62%) reported producing Web-based health information. The vast majority (97.9%) of persons who produced Web-based health information also consumed Web-based health information. There were 622 participants (20.6%) who reported neither consuming nor producing Web-based health information.

### Characteristics Associated With Health-Related Internet Use

Regression analyses incorporating survey sampling weights revealed differences in consumption of Web-based health information according to a number of demographic characteristics. Consumption of Web-based health information decreased with age and increased with education (Table 2). Participants with some college education or more were significantly more likely than persons who did not finish high school to consume Web-based health information (OR 2.24, *P*<.01). Participants with a 4-year college degree or higher had 3.99 times the odds of consuming Web-based health information compared with patients who did not finish high school (*P*<.001). Consumption of Web-based health information was equivalent among persons aged 18-29 and 30-44; after age 45 people were significantly less likely to consume Web-based health information. After adjusting for other covariates, there were no differences in consumption of Web-based health information by race.

Self-reported health status was not independently associated with consuming Web-based health information. However, presence of a chronic condition was associated with significantly more consumption of Web-based health information (OR 1.54, *P*<.01). Having an acute medical exacerbation was also a significant predictor of such consumption; in fact, of all the health-status-related measures, having an acute medical exacerbation was most strongly associated with consuming Web-based health information (OR 2.39, *P*<.001).

**Table 1:** Demographic characteristics, unweighted (N=3014).

		Mean (SD)	n	% (unweighted)
Age		52.6 (19.8)	3014	
**Race**				
	White		2098	69.61
	Black		546	18.12
	Asian/Pacific Islander		85	2.82
	Mixed		80	2.65
	Native American		30	1.00
	Missing		175	5.80
**Sex**				
	Female		1677	55.64
**Insurance**				
	None		396	13.14
	VA/Other		73	2.42
	Medicaid		208	6.90
	Medicare		710	23.56
	Medicaid + Medicare		197	6.54
	Private		1373	45.55
	Missing		57	1.89
**Education**				
	Less than high school		269	8.93
	High school		830	27.54
	Some college		778	25.81
	4-year college or more		1115	37.01
	Missing		22	0.73
**Income**				
	$10,000-20,000		584	19.38
	$20,001-40,000		582	19.31
	$40,001-75,000		604	20.04
	$75,001-100,000		267	8.86
	$100,001-150,000		413	13.70
	Missing		564	18.71
**Health status**				
	Poor/Fair		604	20.04
	Good		1552	51.49
	Excellent		849	28.17
**Chronic condition**				
	Yes		1498	50.32
**Acute medical exacerbation**				
	Yes		609	20.21

**Table 2:** Predictors of consuming any Web-based health information.

		Odds ratio (95% confidence interval)	*P* value
**Sex**			
	Female	2.05 (1.57-2.68)	<.001
**Race** (Ref: White)			
	Black	0.97 (0.70-1.35)	.87
	Asian/Pacific Islander	1.72 (0.84-3.52)	.14
	Mixed	1.44 (0.61-3.42)	.41
	Native American	1.65 (0.29-9.47)	.57
**Education** (ref: less than high school)			
	High school	1.16 (0.66-2.06)	.60
	Some college	2.24 (1.25-4.02)	<.01
	4-year college or more	3.99 (2.24-7.11)	<.001
**Health status** (ref: Poor/Fair)			
	Good	1.13 (0.74-1.71)	.57
	Excellent	1.27 (0.80-2.01)	.32
**Age** (ref: 18-29)			
	30-44	0.80 (0.53-1.22)	.30
	45-64	0.60 (0.41-0.89)	.01
	65+	0.28 (0.18-0.44)	<.001
**Chronic disease**			
	Yes	1.54 (1.13-2.10)	<.01
**Acute medical exacerbation**			
	Yes	2.39 (1.61-3.56)	<.001

### Characteristics Associated With Being a Consumer Versus Producer of Health-Related Internet Content

Having an acute medical exacerbation was significantly associated with producing Web-based health information (Table 3). Persons with an acute medical exacerbation had 1.97 the odds of producing Web-based health information compared to people without an acute medical exacerbation (*P*=.003). In contrast, self-reported health status and the presence of a chronic condition were not significantly associated with being a producer of Web-based health information. Similarly, no demographic variables (including sex, race, age, and education) were associated with producing Web-based health information.

### Types of Web-based Health Information Consumed and Produced by Individuals With and Without Acute Medical Exacerbations

In a post-hoc analysis, we evaluated the type of Web-based health information consumed and produced by participants with and without acute medical exacerbations. In multivariate regression, compared to participants without acute medical exacerbations, participants with acute medical exacerbations were much more likely to seek medical treatment or procedure information, drug safety information, medical test results information, information about drugs advertised, and reviews of providers or treatments (Table 4). Data indicate that regardless of whether patients had acute medical exacerbations, the type of Web-based health information they were most likely to consume was that regarding a disease or medical problem. We found no difference among patients with and without acute medical exacerbations in terms of the source of the Web-based health information they consumed (peer, professional, or both). There were also no differences among patients with and without acute medical exacerbations with respect to the type of Web-based health information they produced (personal health experience vs reviews of providers, hospitals, or treatments).

**Table 3:** Predictors of producing (versus consuming) Web-based health information.

		Odds ratio (95% confidence interval)	*P* value
**Sex**			
	Female	1.07 (0.74-1.55)	.72
**Race** (Ref: White)			
	Black	0.82 (0.50-1.35)	.44
	Asian/Pacific Islander	0.78 (0.26-2.36)	.66
	Mixed	1.44 (0.57-3.63)	.44
	Native American	0.79 (0.21-2.97)	.56
**Education** (ref: less than high school)			
	High school	0.83 (0.35-2.00)	.68
	Some college	0.94 (0.40-2.22)	.89
	4-year college or more	1.10 (0.48-2.55)	.82
**Health status** (ref: Poor/Fair)			
	Good	1.09 (0.62-1.90)	.77
	Excellent	0.80 (0.42-1.54)	.51
**Age** (ref: 18-29)			
	30-44	1.53 (0.89-2.63)	.18
	45-64	1.02 (0.59-1.76)	.93
	65+	0.55 (0.29-1.05)	.07
**Chronic disease**			
	Yes	1.42 (0.91-2.21)	.12
**Acute medical exacerbation**			
	Yes	1.97 (1.26-3.08)	<.01

**Table 4:** Type of consumption among persons with acute medical exacerbations (after multivariate adjustment).^a^

Type of consumption	% of patients with acute medical exacerbations	% of patients without acute medical exacerbations	*P* value
Disease or medical problem information	95.4	92.0	.09
Medical treatment or procedure information	72.3	58.3	.001
Drug safety information (including recalls)	29.3	19.6	<.01
Medical test results information	28.5	16.9	.001
Losing or controlling weight	36.9	35.9	.81
A drug advertised	28.0	19.0	<.01
Reviews of providers or treatments	49.9	40.6	.03

^a^Multivariate regression adjusted for education, health status, chronic conditions, race, age, and sex.

## Discussion

### Principal Findings

Our analyses revealed a number of demographic characteristics associated with consumption of Web-based health information. Females, persons with some college education or more, and persons younger than 44 years were significantly more likely to consume Web-based health information. Conversely, no demographic variable was significantly associated with *producing* Web-based health information. These results corroborate other studies, which have found that Web-based health information-seeking typically increases with education and income and decreases with age [1,17,21,22].

In evaluating health-related variables and consumption and production of Web-based health information, we found having a chronic condition and having an acute medical exacerbation were independently associated with Web-based health information consumption, with acute medical exacerbations being the stronger predictor of this type of consumption. Having an acute medical exacerbation was the only health status–related predictor of becoming a *producer* (rather than a consumer) of Web-based health information. Therefore, having an acute medical exacerbation or a chronic condition is associated with greater engagement with consuming health information technology. For persons who already consumed Web-based health information, having an acute medical exacerbation is associated with becoming a producer of such information.

While over 90% of patients with and without acute medical exacerbations seek Web-based information about health conditions, there are some important differences about the other types of Web-based health information they consume. Persons with acute medical exacerbations in the past 12 months are significantly more likely to seek information regarding medical treatments or procedures, medications, and medical treatment results, and reviews of providers or treatments compared with persons who did not experience an acute medical exacerbation in the past 12 months.

Results indicate that having an acute medical exacerbation was the only significant predictor of producing Web-based health information. This suggests that peer-to-peer health-related Internet content may be skewed towards persons who are sicker. This is important to recognize, given that patients with stable chronic conditions are often consuming this peer-generated information. This overrepresentation of the experiences of persons with acute medical exacerbations may mean there is not enough peer-generated Web-based health information available regarding stable chronic conditions and that the information available may not be applicable to those with stable chronic conditions who wish to prevent an acute medical exacerbation.

Our analysis also revealed the discrepancy between objective evaluations of health and interpretation of one’s own health. The vast majority of our sample had a self-reported health status of good or excellent, yet over half reported having at least 1 chronic condition. Among persons with a chronic condition, 16% reported their health as excellent and half reported their health as good. Among persons with an acute medical exacerbation, 16% reported their health as excellent and less than half reported their health as good. These results suggest that patients are likely to overestimate their own health status.

Having an acute medical exacerbation may be a “window of opportunity” in which health-related online behavior changes. Patients appear to be seeking health information at this time and may be especially receptive to health information provided by their care team. It is also possible that patients are seeking Web-based health information because they are not receiving sufficient information from their providers. The desire for health information around the time of a health status change suggests a role for providers to direct patients to high-quality, easy-to-understand online information, especially information regarding treatments and procedures. This is especially important to provide to patients who have had a recent acute medical exacerbation. These online resources should augment, but not replace, the distribution of printed patient care instructions and information about relevant procedures and treatments.

The fact that patients are both more likely to consume as well as produce Web-based health information around the time of an acute medical exacerbation suggests that this time may also represent a window of opportunity regarding health behavior. Experiencing an acute exacerbation may be a time when patients take more ownership over their health status. There is some cohort-based data to support this. In a study of 253 patients followed one month after an Emergency Department (ED) visit, 12% reported abstaining from smoking for 30 days after the emergency department visit and another 38% reported that they were able to quit smoking for some period of that time. Having a smoking-related emergency department visit was the strongest predictor of abstinence/attempts to quit in these patients [23]. While this indicates that acute medical exacerbations are catalyzing health behavior change, more causal studies are needed to further explore this line of inquiry, as well as what kinds of Web-based and other health-oriented tools may be more helpful and effective at this point.

It may be important to expand the role of the health provider to educate patients not only verbally about their health and medical conditions, but also educate them as to the best Web-based sources of accurate and relevant health information. Not all Web-based health information is considered equal [24]. It can be difficult for patients to identify high-quality versus low-quality information, especially as the accuracy of a website is in fact poorly associated with its credibility [24]. However, routing all patients to the same high-quality website is likely not the optimal solution, as patients have varying abilities in understanding this information. For example, evidence indicates over 80% of post-surgical patients had difficulty understanding Web-based health information and approximately one-third reported that retrieving such information was overwhelming [25].

### Limitations

This analysis was based on previously collected survey data and is subject to limitations. All data were self-reported and may be subject to recall or other bias. Data were collected via mobile phone and landline telephone surveys, and it is possible that persons who choose to respond to such surveys are systematically different from persons who choose to not participate in these surveys. Internet use was assessed as a binary variable, and it is possible the frequency or timing of Internet use varied among survey participants. These data are also cross-sectional, and we are therefore not able to tease out the chronology of events. We found that having an acute medical exacerbation was significantly associated with consuming and producing Web-based health information. It is possible that an acute medical exacerbation led to persons seeking Web-based health information, or that persons used the Internet to search for health information that led them to conclude they were having an acute medical exacerbation for which they sought medical attention.

### Conclusions

This analysis provides new insight into the use of the Internet for health purposes and suggests that having an acute medical exacerbation is a time when patients’ Web-based health behavior changes and patients become more engaged with the Internet for the purposes of heath information. Practice-based implications of this research include providing high-quality Internet health links for patients around the time of an acute medical exacerbation and leveraging the window of opportunity around an acute medical exacerbation to provide patients with online tools to engage health behavior change.
